# Liquid Biopsy for Solid Ophthalmic Malignancies: An Updated Review and Perspectives

**DOI:** 10.3390/cancers12113284

**Published:** 2020-11-06

**Authors:** Arnaud Martel, Stephanie Baillif, Sacha Nahon-esteve, Lauris Gastaud, Corine Bertolotto, Barnabé Roméo, Baharia Mograbi, Sandra Lassalle, Paul Hofman

**Affiliations:** 1 Ophthalmology Department, University Hospital, Cote d’Azur University of Nice, 06 000 Nice, France; martel.a@chu-nice.fr (A.M.); baillif.s@chu-nice.fr (S.B.); nahon-esteve.s@chu-nice.fr (S.N.-e.); 2 CNRS UMR7284, Inserm U1081, Institute for Research on Cancer and Aging Nice (IRCAN), FHU OncoAge, Cote d’Azur University, 06 000 Nice, France; barnabe.ROMEO@univ-cotedazur.fr (B.R.); Baharia.MOGRABI@univ-cotedazur.fr (B.M.); 3 Oncology Department, Antoine Lacassagne Cancer Centre, 06 000 Nice, France; lauris.GASTAUD@nice.unicancer.fr; 4 Centre Méditerranéen de Médecine Moléculaire (C3M), INSERM, Université Nice Côte d’Azur, 06 200 Nice, France; corine.bertolotto@univ-cotedazur.fr; 5 Antoine Lacassagne Cancer Center, FHU OncoAge, 06 000 Nice, France; 6 Laboratory of Clinical and Experimental Pathology, University Hospital of Nice, FHU OncoAge and Biobank BB-0033-00025, IRCAN, 06 000 Nice, France; 7 Laboratory of Clinical and Experimental Pathology, Pasteur 1 Hospital, University Hospital of Nice, 30 voie romaine, 06000 Nice, France

**Keywords:** liquid biopsy, circulating tumor cells, circulating tumor DNA, uveal melanoma, retinoblastoma

## Abstract

**Simple Summary:**

To date, there is no treatment for metastatic uveal melanoma. Identifying its metastatic spread is essential. Liquid biopsy can identify patients at risk of metastatic spread early. Here, we aim to summarize the current knowledge of liquid biopsy in ophthalmic malignant tumors, including uveal melanoma. Our objective is to establish the current state of liquid biopsy in the ophthalmic field, as well as its perspectives and limitations.

**Abstract:**

Tissue biopsy is considered the gold standard when establishing a diagnosis of cancer. However, tissue biopsies of intraocular ophthalmic malignancies are hard to collect and are thought to be associated with a non-negligible risk of extraocular dissemination. Recently, the liquid biopsy (LB) has emerged as a viable, non-invasive, repeatable, and promising way of obtaining a diagnosis, prognosis, and theragnosis of patients with solid tumors. LB refers to blood, as well as any human liquid. The natural history of uveal melanoma (UM) and retinoblastoma (RB) are radically opposed. On the one hand, UM is known to disseminate through the bloodstream, and is, therefore, more accessible to systemic venous liquid biopsy. On the other hand, RB rarely disseminates hematogenous, and is, therefore, more accessible to local liquid biopsy by performing an anterior chamber puncture. In this review, we summarize the current knowledge concerning LB in UM, RB, conjunctival tumors, and choroidal metastases. We also develop the current limitations encountered, as well as the perspectives.

## 1. Introduction

Tissue biopsy is still considered to be the gold standard for establishing the diagnosis of cancer. However, a tissue biopsy is rarely repeatable over time [1], sometimes associated with significant morbidity, and contraindicated in several malignancies [2]. Recently, the Food and Drug Administration (FDA) approved the use of liquid biopsy (LB) as a pertinent diagnosis, prognosis, and monitoring tool [3,4] in non-small cell lung carcinoma (NSCLC) to avoid invasive tissue biopsy in selected cases [5]. Compared to tissue biopsy, LB has indeed the advantages of being non-invasive, collected from multiple body fluids such as blood, urine, saliva, cerebrospinal fluid, and aqueous humor [6]. At molecular diagnosis, LB detects of several biomarkers such as circulating tumor cells (CTC), circulating tumoral DNA (ct-DNA), circulating tumoral RNA (ct-RNA), micro RNA (miRNA), tumor-related exosomes (TREs), and tumor-educated platelets (TEP) [5]. 

Retinoblastoma (RB) and uveal melanoma (UM) are the most common primary intraocular tumors in childhood and adulthood, respectively [2,7]. Conversely to other cancers, a tissue biopsy is usually not readily available in intraocular malignancies. Intraocular biopsy carries the risks of irreversible intraocular damages, tumoral dissemination, and low sensitivity rates, due to the low amount of tissue harvested [8,9]. Therefore, RB and UM are usually treated based on clinical and radiological findings without histological tissue confirmation [10]. This could explain why LB has gained in clinical practice in intraocular malignancies. 

Interestingly, RB and UM highlight two radically opposed LB paradigms. RB is an intraocular tumor encountered in children that develops intraocularly even in advanced stages. Metastasis and relapse are exceptional, and the prognosis is currently excellent [2]. UM is encountered in adult patients, develops intraocularly but early disseminates in the bloodstream. It is estimated that about half of the patients will develop further metastases and will die in the next 24 months despite achieving local control of the tumor [7]. Based on these clinical features, RB is more eligible to an “ocular LB,” whereas UM is more eligible to a “systemic LB.”

This review aims to summarize the current knowledge on LB in solid ocular malignancies, its limitations, and future perspectives. This review will focus on UM, RB, conjunctival malignancies, and choroidal metastases. Intraocular and adnexal lymphomas are excluded from the field of this review.

## 2. Liquid Biopsy in Uveal Melanoma (UM)

UM is the most common primary intraocular tumor encountered in adulthood [7]. UM diagnosis is based on clinical and B-scan ultrasonography findings. Transscleral and intravitreal UM biopsies have proven to be challenging, associated with low positivity rates, intraocular complications, and extraocular tumor spread [11]. To date, the main indication for performing a tissue biopsy is to assess whether the patient is at low or high metastatic risk [12]. Local treatments include proton beam therapy and brachytherapy for small to medium-sized tumors, and enucleation of the eyeball for larger tumors. Local control is achieved in up to 95% of cases, even in larger UM [10]. Synchronous metastatic spread at the time of the primary tumor treatment is an exceptional finding [7]. Despite this, it is established that around one-third to 50% of the patients will experience a metastatic spread in the ten years following the diagnosis highlighting the concept that UM cells escape from the primary tumor very early and remain in dormancy for a while [13,14]. It is still unclear whether the liver is the primary metastatic site of UM. Despite a better molecular understanding, no treatment has shown efficacy for treating UM metastases [15]. When metastatic spread occurs, the overall survival (OS) does not usually exceed 24 months [16]. 

For a few years, attention has been directed to classify UM patients as low or high metastatic risk based on chromosomic and genetic abnormalities. The aim is to detect as early as possible the metastatic spread to include patients in ongoing clinical trials [17]. 

Identifying a reliable biomarker in UM would be of high clinical relevance. The ideal biomarker should be sensitive, specific, non-invasive, and reproducible [5]. Several pathophysiological factors highlight the fact that LB is a very appealing tool in UM [18]: (i) UM disseminates through the bloodstream many months to years before the local treatment, (ii) LB may provide the missing tumor genetic insights of the primary tumor and in-transit metastases, and (iii) given the lack of effective treatment on UM liver metastases, early LB detection of UM spread may improve patient management with a quicker referral of the patient to clinical trials. 

After summarizing the current knowledge on the molecular characteristics of UM, we will develop the different components of venous LB: CTCs, ct-DNA or ct-RNA), non-coding miRNA, TREs and TEPs.

### 2.1. Molecular Characteristics of UM

Around 80% of UM harbor mutually exclusive *GNAQ* and *GNA11* primary driver mutations [19], which makes UM genetics radically different from cutaneous and even conjunctival melanomas [20]. More rarely, other Gα11/Q pathway mutations (*PLCB4* and *CYSTLR2* mutations) have been reported [21]. Activation of the Gα11/Q pathway is thought to promote uveal melanocyte proliferation by activating several intracellular signals involving MAP kinase, β-catenin/YAP, and AKT/mTOR [21]. Despite being of outstanding importance, Gα11/Q pathway mutations are not sufficient to explain UM dissemination. 

UM dissemination and prognosis are more likely related to secondary driver mutations (*BAP1, SF3B1*, and *EIF1AX*) occurring later in the carcinogenesis. Of them, *BAP1* mutations located in chromosome 3 appear to the most meaningful for the clinician [22,23,24]. *BAP1* is a deubiquitinating protease involved in ubiquitin signaling. For unclear reasons, the loss of *BAP1* is associated with a poorer UM prognosis [23]. *SF3B1* and *EIF1AX* are associated with a better prognosis. 

Several chromosomal abnormalities, such as loss of chromosome 3 and 8q gain, are found in high metastatic risk patients [25]. These chromosomal abnormalities result in significant modifications in gene expression profiles (GEP). Recent studies now distinguish low (class 1a), intermediate (class 1b) and high (class 2) UM metastatic risk based on GEP [26]. Interestingly, class 1 UM are often associated with *SF3B1* and *EIF1AX* mutations, whereas class 2 are more frequently associated with *BAP1* mutations [21]. 

Table 1 summarizes the main targetable UM features in LB.

### 2.2. CTCs

**Figure 1:**
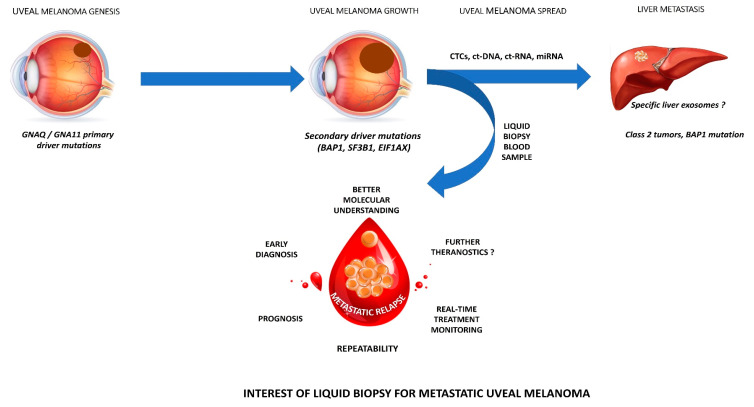
legend: Interest of liquid biopsy for metastatic uveal melanoma.

CTCs were identified for the first time in 1869 by Ashworth, who identified tumor cells in the bloodstream of a freshly dead patient [27]. However, the concept of CTCs detection emerged during the 1990s. CTCs are thought to originate from the most invasive clones of the primary malignancy or its metastases [28]. CTCs may allow a better understanding of the underlying metastatic cascade (Figure 1). Table 2 summarizes the primary studies which investigated CTCs in UM patients. 

#### 2.2.1. CTC Isolation and Identification

In agreement with previous studies on other solid cancers, most UM studies have considered the venous blood for collecting CTCs. Only one study compared the venous versus the arterial way for collecting CTCs and concluded that arterial samples achieved higher CTCs identification rates (100%) compared to venous samples (52.9%) [32]. However, arterial samples can be associated with increased patient’s discomfort and may exceptionally provide complications such as bleeding, ischemia, pseudoaneurysms, and fistula formation [40]. 

CTCs were collected from blood samples (from 7.5 mL to 50 mL) in combination with anticoagulant agents (heparin or EDTA). CTCs process was performed up to 72 h following the collection [29,35]. Immunomagnetic and sizing methods mainly collect CTCs with Cellsearch, which is the only FDA approved CTC collector device [29,31,32,35]. The advantages of the Cellsearch device include reproducibility even if a lack of sensitivity has been highlighted by several authors [5]. Two studies used a ISET (isolation by size of epithelial tumor cells) device [34,36]. 

CTCs may be identified directly or indirectly. Direct identification is allowed by microscopic examination. UM CTCs are defined as single cells or clusters measuring > 16 µm, with an oval shape, a nucleocytoplasmic ratio > 50%, an irregular and hyperchromatic nucleus, and basophilic cytoplasm [36]. Indirect identification is based on the use of multiple antibodies. Most studies have identified UM CTCs by using the high molecular weight melanoma-associated antigen (HMW-MAA), also called Melanoma chondroitin sulfate proteoglycan (MCSP) [18,29,32,35,37,39]. The Cellsearch circulating melanoma cell test also stains the cells with CD45 and CD34 antibodies to rule out leukocyte and endothelial cell detection [35]. Tura et al. reported high CTC detection rates using NKI/C3 and NKI/beteb antibodies [33]. Mazzini et al. used anti-S-100, anti-tyrosinase, and anti-MART1 antibodies following ISET filtration for detecting CTC [34]. Regardless of the method, little is known about the UM de-differentiation process during the metastatic spread. So far, most studies argued that screening a combination of surface markers was associated with a more accurate detection rate (3,4). 

#### 2.2.2. Main Clinical Findings

CTCs’ specificity appears to be reliable. Ulmer et al. were the first in 2008 to investigate CTC detection in UM and healthy patients [39]. By using an immunomagnetic device, they found CTCs in 19 of 52 UM patients (19%), whereas no CTCs were detected among the 20 healthy controls. This result was confirmed by two studies from the same Italian team in 2010 and 2014. Pinzani et al. and Mazzini et al. used the ISET device to detect CTCs in 31% and 55% of their primary and metastatic UM, respectively, while no CTCs were detected in their healthy controls [34,36]. 

Interestingly, CTC detection appears to be useful for distinguishing benign choroidal naevi from a primary or metastatic UM. By using ISET technology, Mazzini et al. were the first to investigate CTCs in 31 UM and ten benign uveal naevi patients [34]. CTCs were detected in 55% of UM patients, whereas no CTCs were found in uveal naevi patients. Similarly, Bande et al. detected CTCs in 4 of 8 UM patients using the Cellsearch device, whereas no CTC was detected in patients with choroidal naevi [31]. To date, no CTC has never been identified in patients diagnosed with a benign choroidal lesion [31,34]. Ophthalmologists are sometimes faced with challenging pigmented choroidal lesions [41], and CTCs could help to distinguish benign from malignant lesions.

CTC detection rate is highly variable in the UM literature. The median number of CTCs detected ranged between 1 [37] and 8 [34], which is relatively low compared to other solid tumors [42]. Suesskind et al., therefore, questioned whether surgical manipulation of the primary UM could disseminate CTCs in the bloodstream [37], by taking blood samples before and 30 min after the surgery. Even if the median CTC count was more important following surgery (7.5 versus 1), CTCs were detected in 14% and 10% of patients before and postoperatively, respectively. Eide et al. found venous CTCs in only 4 of 328 (1.6%) patients by using an immunomagnetic method despite using multiple anti-melanoma antibodies [38]. By contrast, Tura et al. found CTC in 93.6% of their patients by using an immunomagnetic detection method associated with NKI/C3 and NKI/beteb antibodies [33]. Eide et al. and Terai et al. found a better detection rate when harvesting CTCs from the bone marrow and the arterial bloodstream, respectively, compared to the venous compartment [32,38]. CTC detection rate does not seem to be related to the CTC collection method since immunomagnetic and size isolation devices provided almost the same results [31,32,34,36]. Of interest, as outlined by some authors, repeating blood samples over follow-up monitoring by using the same method, leads to increasing CTCs detection rates [29,36]. An increased sensitivity rate could explain this by multiplying the blood samples or developing metastatic spread. Anand et al. demonstrated that CTC detection was higher during the metastatic spread (42%) compared to localized tumors (30%) [29].

The relationship between CTCs detection and UM related clinico-biological features is still controversial. CTC detection was found to be correlated with the diameter and height of the primary tumor [34], with ciliary body invasion known to be a pejorative prognosis factor in UM [39], with Class 2 tumors (high-metastatic risk according to the Harbour classification) [29], and with metastatic liver miliary [35]. By contrast, a lot of studies failed to establish any relationship between CTC detection, clinical and biological features [31,32,33,37]. In accordance with several reports in other solid malignancies, Bidard et al. and Mazzini et al. found that CTC detection was associated with lower disease-free survival (DFS) and overall survival (OS) [34,35]. Monosomy 3 is strongly associated with a poorer prognosis in UM. Interestingly, in 2016 Tura et al. achieved to assess Monosomy 3 status in isolated CTCs by using an immune-Fish isolation technique. Monosomy 3 was found in 23 of 40 (58%) patients and was associated with more advanced TNM stages [33]. CTCs monitoring appears as being a reliable prognostic factor. 

### 2.3. Ct-DNA and ct-RNA

Cell-free DNA and RNA result from cell apoptosis or necrosis and are often found in the bloodstream under physiological conditions [43]. Among cell-free DNA, ct-DNA, and ct-RNA are thought to originate from the primary tumor itself, the CTCs, or the micro or macrometastases [1]. Ct-DNA and RNA have been previously validated in several solid malignancies as a reliable diagnosis, prognosis, and disease monitoring tool [28,44]. Recently, ct-DNA “druggable” mutations have been analyzed for therapeutic purposes [45]. Only a few studies, summarized in Table 3, have investigated ct-DNA and ct-RNA in UM. Even though ct-RNA is more unstable than ct-DNA, ct-RNA was the most circulating nucleic acid studied in UM. 

#### 2.3.1. Ct-DNA and ct-RNA Detection

In all studies, ct-DNA and ct-RNA were extracted from plasma providing from 6 mL to 50 mL of venous blood. The process was based on traditional PCR and RT-PCR techniques. Most studies investigated the tyrosinase and *MELAN-A*/*MART-1* genes [36,46,48,49,50,51,52]. *GNAQ* and *GNA11* are thought to be the driver and highly prevalent mutations in UM [19]. Therefore, Metz et al. and Bidard et al. investigated GNAQ and GNA11 mutations to detect circulating free nucleic acids [35,47]. Keilholz et al. found higher detection rates by screening tyrosinase over MELAN-A/MART-1 and GP100 [52]. Finally, performing multiple PCR in the same sample improved the ct-RNA detection rate [50].

#### 2.3.2. Main Clinical Findings

To date, no study investigated ct-DNA or RNA detection in non-malignant pigmented uveal lesions. Ct-DNA detection in UM ranged from 1.1% [46] to 63% [48]. In accordance with studies in CTCs, multiplying the samples led to higher detection rates. Callejo et al. attained 97% of ct-DNA positivity thanks to 1360 samples performed in 30 UM patients followed-up over a 17-year-period [50]. In agreement with studies in UM CTCs, metastatic patients were found to have higher ct-DNA detection rates compared to non-metastatic patients [52]. To date, no study found a relationship between ct-DNA detection and the intraocular tumor size or height. Two studies investigated whether the treatment of the intraocular tumor could influence the detection of ct-DNA. Boldin et al. identified ct-DNA in 16 of 41 (39%) patients preoperatively. During the postoperative follow-up, ct-DNA was no longer detectable in 69% of the previously positive patients [51]. Charitoudis et al. performed a venous blood sample before and 30 min after the surgical treatment of the intraocular tumor and failed to identify any ct-DNA release [46]. 

Not surprisingly, given its role in tumor dissemination, Bidard et al. demonstrated that both CTCs and ct-DNA detection were associated with liver miliary metastatic spread [35]. Ct-DNA was found to be strongly associated with reduced Progression-Free Survival (PFS) and Overall Survival (OS) [35,36,47,48,49,51]. Metz et al. reported that ct-DNA detection was associated with larger liver metastases [47]. Bidard et al. aimed to compare ct-DNA and CTCs detection in 40 metastatic UM patients. They monitored CTC by using the Cellsearch device and ct-DNA by screening three different GNAQ and GNA11 mutations. In their univariate analysis, both CTCs and ct-DNA were associated with metastatic spread, PFS, and OS. However, only ct-DNA was found to be correlated with PFS and OS in the multivariate analysis. Further studies with larger samples comparing CTC and ct-DNA are warranted before drawing any conclusion. CTCs and ct-DNA advantages and disadvantages in LB are shown in Table 3.

### 2.4. Non-Coding RNAs

There are two types of non-coding RNA based on their size: Long and short non-coding RNA, both of which are involved in gene expression regulation. 

Micro-RNA (miRNA) are small (around 22 nucleotides) non-coding RNAs molecules found in the tissues and the bloodstream [53]. More than 1500 miRNA have been identified in humans. They are involved in the regulation of several biological processes, including post-transcriptional gene expression and cell communication. MiRNA act by adding post-transcriptional support in the 3′ untranslated region (3′UTR) of their targeted mRNA [54]. 

#### 2.4.1. Non-Coding RNAs Detection

MiRNAs can be detected in tissue biopsy samples and many body fluids, and thus, would constitute a relevant cancer biomarker. MiRNA can be isolated alone in the plasma (cell-free miRNA) or encapsulated in small vesicles (exosomes) by using RT-PCR, microarray, and deep sequencing [53]. 

Long non-coding RNAs (lncRNA) are a subcategory of RNA with a size larger than 200 nucleotides that do not encode proteins. Plasmatic LncRNAs have been incriminated in several malignancies by modulating the transcriptional and post-transcriptional processes.

#### 2.4.2. Main Clinical Findings

Growing evidence has incriminated miRNAs as being a significant cancer player by upregulating several oncogenes [55]. In UM, miRNAs have been found to be an interesting tool for the diagnosis and prognosis of UM [54]. Several studies aimed to identify specific UM miRNA clusters [56]. Stark et al., by collecting serum from 65 consecutive localized and metastatic UM patients, found a panel of 6 miRNAs (miRNA-16, miRNA-145, miRNA-146a, miRNA-204, miRNA-211, and miRNA-363-3p) as being useful for distinguishing benign uveal naevi from UM with a sensitivity and specificity of 93% and 100%, respectively [57]. They also found that miRNA-211 was significantly more expressed in metastatic UM compared to localized UM. Low circulating miRNA-204 was the only miRNA associated with a poorer OS. Based on the literature, miRNA-146a appears as being the most discriminant miRNA for diagnosing UM [57,58,59,60]. In their study conducted in 14 localized UM patients, Russo et al. found that among 754 mi-RNAs tested, mi-RNA-146a was significantly overexpressed in histological samples, as well as in venous blood of UM patients [61]. Interestingly, Ragusa et al. also found mi-RNA-146a being overexpressed by three folds in vitreous samples and vitreous exosomes [58]. Mi-RNA-146a has been previously incriminated in other malignancies, such as papillary thyroid cancer, hepatocellular carcinoma, or leukemia [62]. Mi-RNA-146a has also been involved in the pigmentation and survival of UM cells [63]. However, other studies did not support mi-RNA-146a as being a useful blood biomarker [56,64]. Other mi-RNAs have been incriminated in the metastatic spread of UM: mi-RNA-199a [65], mi-RNA-34a [66], mi-RNA-34b [67], among others. It is still unclear how mi-RNAs biologically induce UM growth and dissemination. Mi-RNA-34a is thought to inhibit the oncogene c-Met, and several studies found decreased mi-RNA-34a rates in metastatic UM [66,67]. Similarly, mi-RNA-137 was found to be dramatically reduced in UM. Mi-RNA-137 is known to play a role in the cycle cell arrest [68] and downregulates the MITF and c-Met oncogenes [69]. In cutaneous melanoma, the downregulation of MITF is known to induce the differentiation and invasiveness of tumor cells [70]. Of interest, it should be noted that the different studies shared only a few mi-RNA, and huge disparities regarding the biological effect of a given mi-RNA have been found [54]. This highlights the complicated interactions of the mi-RNAs with several pathways and the lack of consensus regarding mi-RNA extraction and processing.

Several in vitro studies demonstrated that lncRNAs played a role in UM tumorigenesis. The lncRNA PVT1 (Plasmacytoma variant translocation 1 gene) was found to be overexpressed in tissue biopsy of 28 UM patients [71]. This finding was consistent with those of Xu et al., who investigated the expression of lncRNA PVT1 by using deep-RNA sequencing data from the UM Cancer Genome Atlas. They found that lncRNA PVT1 was overexpressed in about 75% of primary UM and significantly associated with epithelioid UM, extrascleral extension, and poorer overall survival [72]. Of targets, It is postulated that lncRNA PVT1 acts by repressing mi-RNA 17-3, which interacts with MDM2 and p53 proteins [71]. By promoting autophagy, lncRNA ZNNT1 has also been associated with in vitro UM cell death [73]. Other lncRNAs such as lncRNA FTH1P3 [74], lncRNA MALAT1 [75], or HOXA11-AS [76] have also been incriminated in UM tumorigenesis. However, studying lncRNA in UM is limited by many incriminated lncRNAs and related mi-RNAs [77]. To date, no lncRNA has been established as a reliable UM biomarker.

### 2.5. Tumor-Related Exosomes (TREs)

#### 2.5.1. TRE Detection

Exosomes are nano-sized extracellular vesicles released by all cell types, including cancer cells [53]. These nanovesicles are thought to participate in tumor growth by acting on the tumoral micro-environment [1]. They are also incriminated in the development of micrometastases niches [78] and in the late UM metastatic spread [79]. As shuttle, exosomes encapsulate proteins, as well as RNA (ct-RNA) and other tumor-related genetic material such as Ct-DNA, and mi-RNA. Exosomes protect these nucleic acids from degradation in blood, and other biological fluids, which explains their growing interest. Exosomes can be isolated by centrifugation, density, or immune methods. Of interest, the mutational status of certain cancers has been established based on ct-DNA contained in circulating exosomes [53].

#### 2.5.2. Main Clinical Findings

Ragusa et al. found similarities between tumor mi-RNA and exosomal mi-RNA, providing from the vitreous cavity of UM patients [58]. Interestingly, mi-RNA-146a was found to be upregulated in both the vitreous cavity and plasma of UM patients [58]. In their study in metastatic UM, Eldh et al. isolated Melan-A exosomes in liver perfusate during isolated hepatic perfusion [79]. They also found higher total exosome concentration in the venous blood of metastatic UM patients compared to healthy controls. Other studies tried to identify specific TRE panels in UM. In other solid malignancies, exosomes were found to be efficient for distinguishing benign from malignant neoplasms [53]. Further studies focusing on the ability of exosomes to distinguish benign from malignant choroidal pigmented lesions are warranted. As in prostatic cancer, establishing a panel of exosomes could better predict the prognosis and metastatic risk of UM [1].

### 2.6. Tumor-Educated Platelets (TEPs)

Platelets originate from the precursor megakaryocyte cell in the bone marrow and are the second most common cells found in the bloodstream. Several large sample-sized cohort studies evidence that antiplatelet medications were associated with a decreased metastatic spread in several cancers, and inversely, high platelet counts were found in disseminated cancers [80,81]. Based on these indirect correlations, the role of platelets in cancer dissemination has gained popularity. Platelets are thought to crosstalk with the tumoral cells and microenvironment via the release of extracellular vesicles [82]. In turn, cancer cells can incorporate their genetic material such as RNA within the platelets leading to their “education.” As a result, platelets may harbor specific tumoral RNA signatures and may represent a widely available cancer biomarker. TEPs are very stable compared to free circulating nucleic acids and are available in a great amount. Best et al. demonstrated that screening platelet RNA distinguishes cancer patients from healthy donors [83]. TEPs may also be useful for screening and monitoring anti-tumoral targeted therapies. To date, no study investigated TEPs in UM.

### 2.7. Future Perspectives: Towards a Better UM Understanding?

Despite a better understanding of UM over the past decades, two questions have not yet been resolved: (i) How can UM relapse a few years after the diagnosis despite achieving local control of the disease? (ii) Why is the liver the primary metastatic site? LB may be of prime interest to investigate these unsolved questions.

UM relapse is thought to be based on the concept of dormancy. Tumor dormancy is defined as a biological phenomenon in which CTCs remain quiescent and undetectable in a unique and specific microenvironment [84]. For unexplained reasons, these in dormancy cells could be reactivated and will further disseminate to their metastatic site of preference. Several studies showed that UM metastatic spread follows a bimodal curve with early- and late-onset [85]. Late metastatic relapse could be explained by rapid growth and dissemination of previously in dormancy UM cells. Eide et al. conducted in 2009 a pilot study in 328 primary UM patients undergoing peripheral blood and bone marrow samples at the time of the primary eye treatment (6). UM tumor cells were isolated by using an immunomagnetic method associated with several anti-melanoma antibodies. Tumoral cells were found in 1.6% and 29.9% of patients in blood and bone marrow, respectively. The authors hypothesized that peripheral blood could be considered as a transport medium and bone marrow as the reservoir for further in dormancy UM cells. As outlined above, TEPs provide from the bone marrow. One might hypothesize that bone marrow UM cells could interact with the platelets in their primary production site to become TEPs. The latter will be further released within the bloodstream and could trigger proliferation and dissemination messages to the tumoral in dormancy bone marrow UM cells. However, Eide et al. published in 2015 and 2019, the follow-up and overall survival of their 328 patients and failed to identify bone marrow involvement as a pejorative prognosis factor (35,36). Surprisingly, bone marrow involvement was more common in early TNM stages, highlighting the possibility that more aggressive cancer cells may have lost their surface epitope by a de-differentiation process leading to an underestimation of bone marrow micrometastases. In a subgroup analysis, they found that metastases were more prevalent in patients harboring a low number of bone marrow melanoma cells. The authors hypothesized that, for unknown reasons, most UM cells found in the bone marrow were apoptotic and that only a few UM cells will further develop and spread.

To date, it is still unclear whether UM metastasizes predominantly to the liver. Some authors demonstrated that exosomal integrins could be incriminated in cancer dormancy by guiding the CTCs to their specific niche and in metastatic dissemination by guiding the CTC to the targeted metastatic organ [86]. Of interest, Peinado et al. demonstrated that melanoma exosomes educated bone marrow progenitor cells [87]. Further investigations regarding the connections between TREs and TEPs in the bone marrow microenvironment would be of great clinical relevance.

### 2.8. Limitations

Table 4 summarizes the main limitations encountered with current LB techniques in UM. The most important limitation is the lack of consensus regarding the most appropriate method for isolating and revealing the CTCs. Only two studies compared different liquid biopsy techniques in UM [35,36]. Only a few amounts of CTC have been identified in the current literature by using mainly immunomagnetic isolation techniques. This could reflect a low bloodstream shedding or a lack of sensitivity with the isolation CTC devices used. Recent significant improvements have emerged regarding CTC isolation technology. New promising devices based on microfluidics [88] could improve CTC detection rates and need further investigations in UM.

It is still unclear whether circulating UM cells undergo a de-differentiation during the metastatic spread [89] as encountered in several carcinomas with the Epithelial-Mesenchymal Transition (EMT) [90,91]. A study conducted in a UM xenograft model highlighted that numerous transcriptional gene modifications, including the expression of Melan A, occurred during the metastatic process [92]. Therefore, screening multiple genes or surface markers is warranted in CTCs and ct-DNA detection.

### 2.9. Conclusion

LB appears to be a non-invasive and particularly useful approach for detecting and studying UM. Blood LB is of great relevance for distinguishing benign from malignant pigmented choroidal lesions, for detecting relevant mutational status when a tissue biopsy is not available, and has been associated with PFS and OS in several studies. However, LB is faced with a lack of consensus regarding the pre and post-analytical processes. Further studies are warranted to determine whether LB may emerge as a viable biomarker that could be used in clinical practice.

## 3. Retinoblastoma (RB)

RB is the most common primary intraocular malignancy in childhood. RB arises from the photoreceptors located in the inner retinal layers. RB is the result of a mutation of the tumor suppressor gene RB1 located on chromosome 13q [24]. Non-inherited forms are usually unilateral, whereas bilateral or trilateral (pineal gland involvement) RBs are mainly encountered in inherited forms. RB is usually diagnosed when the patient is approximately two years old [2]. Leukocoria and strabismus are the most common clinical signs leading to the diagnosis. Tissue biopsy is usually contraindicated since it is thought to favor extraocular dissemination. Several differential diagnoses such as Coats disease, persistent fetal vasculature, retinopathy of prematurity, coloboma, and toxocariasis may be misdiagnosed as an RB despite using optical coherence tomography and B scan ultrasonography [2]. Studies on enucleated eyes also found significant somatic copy number alterations such as gains on 1q, 2p, 6p, and losses on 13q and 16q [93]. Treatment is not consensual and is based on the laterality involvement and the TNM classification. Localized intraocular RB can be treated by cryotherapy or laser therapy. Intravitreal seeding can now be safely treated by intravitreal injection of chemotherapy medications [94]. More advanced cases may be treated by intraarterial chemoembolization or enucleation. Rarely, retinoblastoma may spread locally into the orbit and the brain through the optic nerve and disseminate to the bone marrow and later to visceral organs [2]. Despite this, the prognosis is usually excellent [95].

Tissue biopsy in RB is relevant for two reasons. Firstly, the biopsy allows for diagnosis confirmation. Differencing RB from Coats disease appears as particularly challenging, and some infants may undergo an enucleation for diagnosis purposes [96]. Secondly, the tissue sample allows the assessment of Rb1 mutational status for prognostic counseling [95]. However, RB biopsy is contraindicated, due to the fear of extraocular tumor seeding [2], and the rate of enucleation has been dramatically reduced thanks to eye-sparing strategies [94]. Therefore, LB has emerged as a possible useful diagnostic and monitoring tool.

In contrast to UM, the hematogenous spread is rarely encountered in RB, and the aqueous humor (AH) sample has gained interest [93]. AH may provide diagnosis, genetic, prognosis, and treatment response data. AH puncture is an easy, relatively non-invasive, and safe procedure performed under general anesthesia in infants. AH sampling may be combined with eye examination performed under general anesthesia in infants, as well as in combination with intravitreal delivery of chemotherapy. Berry et al. demonstrated that a higher AH somatic chromosomal copy number alteration, including 6p gain, was predictive of more advanced and aggressive RBs [97,98]. They found that AH ct-DNA was concordant with ct-DNA providing from a tissue sample of enucleated patients. The same team demonstrated that AH provided a higher ct-DNA sensitivity compared to the blood sample [6]. Gerrish et al. also identified ct-DNA in AH samples of 12 RB patients [99]. AH ct-DNA profile was identical to this found in enucleation samples. Interestingly, a lower quantity of AH ct-DNA was found in patients treated by intravitreal injection of chemotherapy. In recent years, the rate of enucleation for the treatment of RB has been dramatically reduced, limiting tissue analysis to establish the mutational status of the RB1 gene. Kothari et al. recently demonstrated that circulating plasma DNA was able to assess RB1 mutation status non-invasively without the need for biopsy or enucleation [100].

Other AH contents have been investigated, but most of them are limited by their lack of specificity and the lack of genetic status assessment. AH LDH was found to be increased in locally advanced retinoblastoma compared to healthy controls. However, AH LDH did not correlate with the clinical features, the treatments underwent, and serum LDH. In addition, LDH is not specific and may be elevated in glaucoma (sometimes encountered in locally advanced RB) and Coats disease, which is a challenging differential diagnosis [101]. Some studies investigated the AH detection of Neuron Specific Enolase (NSE) secreted by numerous neuroendocrine tumors. These studies found that NSE was increased in enucleated RB eyes compared to controls but failed to demonstrate a correlation with clinical and pathological features [101]. Survivin and TGF-β were found to be elevated in AH and serum of patients with RB with high sensitivity and specificity rates [93]. Unlike the previous biomarkers, they were positively correlated with the clinical and pathological features, especially optic nerve invasion [93,101]. Further studies on these biomarkers are warranted.

Serum biomarkers have been less investigated. Beta et al. demonstrated that several serum miRNAs were up and downregulated in 14 RB patients [102]. They found that miRNA-17, miRNA-18a, and miRNA-20a were upregulated and could be considered as potential biomarkers [102].

The development of AH puncture has led to reconsider the rule, which stated that an RB eye should never be violated. Since tissue biopsy is still contraindicated, retinoblastoma research is now directed to AH analysis for establishing RB diagnosis, prognosis, and treatment response monitoring. Relevant and large sample sized studies are currently ongoing to assess the indications of AH puncture and to determine the best biomarker.

## 4. LB in Conjunctival Malignancies

Conjunctival melanoma and squamous cell carcinoma are rare ocular neoplasms. Contrary to intraocular malignancies, the biopsy is routinely performed in conjunctival tumors to confirm the diagnosis and identifying key mutational status [103]. Taken together, this could explain why LB has been little investigated in conjunctival tumors.

Conjunctival melanoma accounts for less than 5% of ocular tumors but is associated with a mortality rate of around 30% [104]. Despite its location, conjunctival melanoma behavior is more related to cutaneous melanoma rather than uveal melanoma [20]. Unlike UM, conjunctival melanomas disseminate through lymphatics and hematogenous routes, harbor *NF1, BRAF, NRAS*, and *KRAS* mutations [103], and may be treated with targeted therapies [105] and immunotherapies [106,107]. LB has not been studied specifically in conjunctival melanoma. However, one might hypothesize that LB techniques developed in cutaneous melanoma may be relevant in conjunctival melanoma [108].

Conjunctival squamous cell carcinoma is an exceptional ocular surface malignancy with an incidence of 2–35 per million [109]. Conjunctival carcinoma is strongly related to UV exposition and HIV. Recently, HPV infection has been incriminated in conjunctival carcinoma development [110]. Dissemination may be local to the orbit, lymphatic, and/or hematogenous. Treatment involves surgery sometimes associated with topical chemotherapy and/or radiation beam therapy [111]. To date, there is no standard of care for metastatic conjunctival carcinoma.

## 5. LB in Choroidal Metastases

Although being often asymptomatic, choroidal metastases are the most common intraocular malignancies in adulthood [112]. Breast and lung carcinomas are the most common primary tumor encountered [112]. The increasing overall survival seen in several malignancies, including breast and lung cancers, has led to a higher detection rate of choroidal metastases [112]. In most cases, choroidal metastases occur at a late stage of the disease, and LB does not appear to be useful. LB may be relevant in the case of (i) unknown primary malignancy, (ii) difficulty for performing the biopsy of the primary tumor, and (iii) if the patient underwent multiple malignancies. Choroidal metastases may be the first clinical manifestation of the underlying malignancy in about one-third of patients [113,114]. In their study conducted in 420 and 96 patients experiencing choroidal metastases, Shields et al. [114] and Konstantidinis et al. [113] did not know the primary tumor in 34% and 28% of their patients, respectively. Several patients benefited from an intraocular biopsy to identify the primary tumor. This biopsy may be risked and associated with a lack of sensitivity, given the low amount of tissue available [8]. LB may be a particularly useful and non-invasive method for identifying the primary tumor. Our team recently reported a patient with bilateral choroidal metastases without a known primary tumor. Plasma ct-DNA providing from a Non-Small Cell Lung Carcinoma (NSCLC), was found. In addition, EGFR mutation was identified, and targeted therapy was successfully initiated [115]. Aqueous humor analysis may also provide new insights. Daxecker et al. reported 40 years ago, a case of elevated anterior chamber CEA in a patient with bilateral choroidal metastases from breast carcinoma [116].

## 6. Conclusions

LB is a non-invasive and promising technique for diagnosing and monitoring intraocular malignancies. LB may be useful in daily clinical practice to (i) confirm the cancer diagnosis without tissue biopsy, (ii) to establish a reliable prognostication, (iii) to allow early detection of metastatic spread, and (iv) monitoring treatment response. LB may also provide new pathophysiological insights concerning tumor dissemination and dormancy. Although very promising, LB suffers from several inherent limitations. To date, there is a lack of consensus regarding the ideal biomarker. Pre- and post-analytic processes differ widely from a study to another, limiting their reproducibility. The biggest challenge will be to establish an international consensus among the ocular oncology centers.

## Figures and Tables

**Table 1:** main targetable antibody and molecular markers of uveal melanoma.

Antibody Marker	Molecular Marker
Melan-A	Tyrosinase
HMW-MAA	GNAQ, GNA11
GP 100	BAP 1

**Table 2:** Summary of the main studies investigating circulating tumor cells (CTCs) in uveal melanoma (UM).

Authors	Study Population	Number of Patients	CTC Isolation Method and Device	CTC Identification	Mean CTC (Range)	Main Findings	Follow-Up: Months (Range)
Anand et al. [29]	Primary and metastatic UM	39 patients 20 primary UM 19 metastatic UM	Immunomagnetism Cellsearch	Cellsearch protocol: DAPI+ HMW-MAA+ CD146+ CD45- CD34-	5.9 (1–38)	At initial sampling: CTC detected in 14 out of 39 (36%) patients. CTC detected in 6/20 (30%) primary UM and 8/19 (42%) metastatic UM During the follow-up period: CTC detected in 21/39 (54%) of patients CTC were more likely detected in Class 2 UM (83%)	16.4
Tura et al. [30]	Primary UM	44 UM patients	Immuno-FISH isolation	NKIC3 and MCSP antibodies	Median: 2.4 (0–10.2) Median CTC in Monosomy 3 patients: 3.4 (0.7–10.2) Median CTC without Monosomy 3: 1.2 (0.3–8.4)	CTC detected in 40/444 (91%) patients Monosomy 3 detected in 23/40 (58%) patients Monosomy 3 on CTC associated with a higher TNM stage (T3)	48
Bande et al. [31]	Primary UM Uveal naevi	12 patients 8 primary UM 4 uveal naevi	Immunomagnetism CellSearch	Cellsearch protocol: DAPI+ HMW-MAA+ CD146+ CD45- CD34-	UM: 1 (0–3)	CTC detected in 50% of UM patients and 0% in uveal naevi No relationship between CTC detection and the UM clinical-pathological features	25 (16–27)
Terai et al. [32]	Metastatic UM	17 patients 10 hepatic metastases 7 extra hepatic metastases	Immunomagnetism CellSearch	Cellsearch protocol: DAPI+ HMW-MAA+ CD146+ CD45- CD34-	Arterial: median: 5 (1–168) Venous: median: 1 (0–5)	No morphological difference between CTC collected through the arterial and venous route Arterial blood: CTC detection in 100% of cases Venous blood: CTC detection in 52.9% of cases No correlation between CTC number and number and size of metastases	None
Tura et al. [33]	Primary UM	31 patients	Immunomagnetism Immunobeads	2 antibodies: NKI/C3, NKI/beteb	Median: 3.5 (0–10.2)	CTC detected in 29/31 (93.6%) of patients No correlation between the CTC count and clinical parameters	None
Mazzini et al. [34]	Primary UM Metastatic UM Uveal nevi	31 UM 10 uveal nevi	Isolation by size ISET	Antibodies anti S100, anti MART-1 and anti-tyrosinase	Median 8 (2–50)	CTC detected in 17/31 (55%) of UM patients. No CTC detected in uveal nevi patients No correlation between clinical and biological parameters and CTC positivity Detection of >10 CTC associated with a larger basal diameter, tumor height, disease free survival, and OS	24–60
Bidard et al. [35]	Metastatic UM	40 patients	*For CTC detection:* Immunomagentism Cellsearch *For Ct-DNA detection:* BiPAP technique with 3 mutations screening: GNAQ c.626A > T, GNAQ c.626A > C and GNA11 c.626A > T	Cellsearch protocol: DAPI+ HMW-MAA+ CD146+ CD45- CD34-	0 CTC: 70% ≥ 1 CTC: 30% *1 CTC: 10%* *3 CTC: 15%* *12 CTC: 2.5%* *20 CTC: 2.5%* DNA quantity: Median: 4.1 ng/mL (0.5–512)	Liver miliary associated with higher ct-DNA levels and CTC counts Correlation between CTC, ct-DNA, and tumor volume assessed by liver MRI Univariate analysis: CTC and ct-DNA positivity associated with PFS and OS Multivariate analysis: Only ct-DNA was associated with PFS and OS	8 (median)
Pinzani et al. [36]	Primary UM Healthy Controls	41 primary UM 16 controls	mRNA detected by RT-PCR (41 patients) CTC: Isolation by size using ISET device (16 patients) Blood samples repeated every 6 months	CTC morphology: cell size > 16-micron, nucleocytoplasmic ratio > 50%, irregular nuclear shape, hyperchromatic nucleus, and basophilic cytoplasm	PCR: median: 0.8 cell equivalent /mL of blood (0.1–14.4) ISET: 5.8, 2.33, 2.00, 1.25, and 0.75 CTC/ml	RT-PCR positivity in 20/41 (49%) of patients among at least one of the blood samples PCR positivity associated with decreased PFS and OS CTC detected in 5/16 (31%) patients Tyrosinase level correlated with CTC detection	55
Suesskind et al. [37]	Primary UM	81 primary UM 94 samples before /after treatment	Immunomagnetism MACS	MCSP antibody	Preoperative median CTC count: 1 (1–8) Post-treatment: median CTC count: 7.5 (1–26)	CTC count before and after treatment (enucleation =7, radiotherapy stereotaxic =49, endoresection =19, brachytherapy =15, thermotherapy = 4) Before treatment: CTC detected in 13/94 (14%) of patients After treatment: CTC detected in 9/94 (10%) of patients No significant difference in terms of the CTC count before and after treatment No relationship between the CTC positivity and patient characteristics and metastatic status	16 (median)
Eide et al. [38]	Primary UM	328 patients	Immunomagnetism	Several anti-melanoma antibodies (9.2.27 antimelanoma-associated antibody, IgG1 Ep-1 antibody, 376.96 antibody)	Median cells number: 50 (1–500)	CTC detected in 4/328 (1,6%) patients Tumor cells detected in 98/328 (29.9%) patients in bone marrow No relationship between bone marrow tumor detection and further metastatic spread	60
Ulmer et al. [39]	Primary UM Healthy controls	52 primary UM before treatment 20 healthy controls	Immunomagnetism MACS	MCSP antibody	Median: 2.5 (1–5) for 50 ml	CTC detected in 10/52 (19%) of patients No CTC detected in controls CTC positivity associated with ciliary body invasion, advanced local tumor stage, and anterior tumor localization Multivariate analysis: Only ciliary body involvement associated with CTC positivity	None

NR, not reported; DAPI, 4′,6-diamidino-2-phenylindole; HMW-MAA, human high molecular weight-melanoma-associated antigen; OS, overall survival; PFS, progression free survival; MCSP, melanoma chondroitin sulfate proteoglycan; MACS, magnetic activated cell sorting; ISET, isolation by of epithelial tumor cells.

**Table 3:** Summary of the main studies investigating ct-DNA and ct-RNA in uveal melanoma.

Authors	Study Population	Number of Patients	Ct-DNA/ct-RNA Detection	Main Findings	Follow-Up: Months (Range)
Charitoudis et al. [46]	Primary UM undergoing surgery	202 patients	RT-PCR screening tyrosinase and MELAN-A/MART-1	RT-PCR tyrosinase positive in 2/184 (1.1%) patients before and 4/180 (2.2%) patients after surgery RT-PCR MELAN-A/MART-1 positive in 20/184 (10.9%) before and in 25/180 (13.9%) patients after surgery RT-PCR results on MELAN-A/MART-1 and Tyrosinase levels were not affected by surgical manipulation	24
Metz et al. [47]	Primary and metastatic UM	28 patients	PCR screening GNAQ Q209 (298 bp), GNAQ R183 (212 bp), GNA11 Q209 (150 bp), and GNA11 R183 (249 bp)	Oncogenic GNAQ/GNA11 mutations identified in ct-DNA of 9 out of 22 (41%) metastatic patients. Ct-DNA correlated with the metastatic status ct-DNA detected in younger patients with larger metastases	None
Schuster et al. [48]	Metastatic UM	68 patients	RT-PCR screening tyrosinase and MELAN-A/MART 1	RT-PCR positive in 43/68 (63%) patients 31 patients positive for tyrosinase 40 patients positive for MELAN-A /MART 1 28 patients positive for both RT-PCR positivity associated with poorer PFS and OS	10 (median)
Schuster et al. [49]	Primary UM	110 patients	RT-PCR screening tyrosinase, MELAN-A/ MART1	RT-PCR positive in 11/110 (10%) patients (5 tyrosinase, 5 MALAN-A/MART1, 1 both) No correlation between RT-PCR positivity and clinical features Univariate analysis: The relationship between RT-PCR positivity and time to progression and OS RT-PCR positivity indicated an increased risk of metastasis and disease-specific mortality	22 (median)
Callejo et al. [50]	Primary UM	30 patients	RT-PCR screening tyrosinase, Melan-A	RT-PCR positive in 29/30 (97%) patients (119 visits, 1360 samples, 2720 PCR performed) No correlation between RT-PCR positivity, tumor size and treatment	NR
Boldin et al. [51]	Primary UM	41 patients	RT-PCR screening tyrosinase	RT-PCR positive in 16/41 (39%) patients at baseline 11/16 (69%) patients initially positive were negative after treatment RT-PCR positivity associated with decreased 5-year OS RT-PCR positivity not correlated with tumor size and histology	60–66
Keilholz et al. [52]	Primary and metastatic UM	61 patients 21 primary UM 40 metastatic UM	RT-PCR screening tyrosinase, MELAN-A/MART-1 and GP100	Primary UM: tyrosinase detected in 3 (12.5%) patients, MELAN/MART detected in 1 (4%) patient and GP100 detected in 1 (4%) patient. Metastatic UM: Tyrosinase detected in 24 (60%) patients, Melan/MART 31 (77%) patients and GP100 in 4/26 (15%) patients GP100 positive in 4/40 (10%) samples. Accuracy detection rates: Tyrosinase > Melan > GP100	6

RT, reverse transcriptase; OS, overall survival; PFS, progression free survival; NR, not reported.

**Table 4:** Advantages and limitations of liquid biopsies for uveal melanoma.

LB Feature	Advantages	Disadvantages
CTC	● Allows a better understanding of the metastatic process by screening genetical mutations and surface biomarkers ● Allows laboratory cell culture and further in vivo investigations	● Lack of consensus concerning pre- and post-analytic processes ● May be less reliable than ct-DNA, according to Bidard et al.
Ct-DNA	● More reliable and standardized techniques compared to CTC ● More stable than ct-RNA	● Less instructive than CTC in understanding the underlying tumorigenesis ● GNAQ and GNA11 mutations are not found in all UM
Ct-RNA	● Detection by reliable techniques (RT-PCR)	● Instability (degradation by RNAase) Low abundance ● Half-life very low
miRNA	● Longer half-life, especially when encapsulated ● More stable compared to ct-DNA and ct-RNA ● Detected by reliable techniques (RT-PCR)	● Lack of consensus regarding pre- and post-analytic processes ● Conflicting results regarding the role of certain mi-RNAs
TRE	● Stable ● Long half-life ● Possibility to investigate mi-RNA, DNA, RNA, as well as surface markers	● Lack of consensus regarding exosome definition (different definitions based on the size to distinguish exosomes from other small extracellular vesicles) ● Lack of available studies ● Lack of process standardization
TEP	● Promising preliminary results in other solid malignancies ● TEPs are easily obtained and processed ● Available in large amounts	● Lack of studies into UM
